# Editors' note

**DOI:** 10.4103/0974-2069.41048

**Published:** 2008

**Authors:** Bharat Dalvi, Raman Krishna Kumar

It gives us great pleasure in presenting to you, on behalf of the Pediatric Cardiac Society of India (PCSI), the inaugural issue of the Annals of Pediatric Cardiology

Pediatric cardiology and cardiac surgery have made great strides in the past three decades. The morbidity and mortality associated with congenital heart disease has dramatically improved. This is due to advances in imaging technology, monitoring systems in the OR and CCU, interventional modalities, management of cardiopulmonary bypass as well as surgical perspective and approach. Much progress has also been made by our colleagues in anesthesiology, intensive care, nursing, physiotherapy, microbiology, fetal medicine and genetics. This multidisciplinary approach has been at the center of the success story of our specialties.

The Annals of Pediatric Cardiology is being launched with an idea to offer a common platform to members of all these subspecialties to come together to express their views and showcase their data. Scientific material may be presented in this journal as an original article, brief communication, case report, images, review, ‘how I do it’ or as an invited editorial. We also plan to have debates (point-counterpoint), hemodynamic rounds and summary of expert opinions on controversies, which would be helpful to trainees and fellows. The pattern of our Journal will be flexible and may evolve depending on the feedback we receive from our readers and advisors.

Through the window of this Journal we also wish to look a little beyond ‘pure science’. We welcome contributions pertaining to challenges of medical education, postgraduate training, retaining talent, new program development, balancing economics with patient care in the developing world and any other ‘personal point of view’ that directly or indirectly affects our patients and their families.

Our first issue is an example of what is to be expected from this Journal in the time to come. We look forward to your scientific contribution as well as feedback (preferably more of critical brickbats and less of flowery bouquets!!) to enable us to improve in our efforts. This venture was possible due to help and encouragement from our colleagues on the Editorial and Advisory Boards, contributors (of the scientific articles), reviewers and financial supporters. We also acknowledge unstinted support from our Associate Editors and the core committee of the PCSI.

Happy reading!

